# Validation of Blood Volume Fraction Quantification with 3D Gradient Echo Dynamic Contrast-Enhanced Magnetic Resonance Imaging in Porcine Skeletal Muscle

**DOI:** 10.1371/journal.pone.0170841

**Published:** 2017-01-31

**Authors:** Stefan Hindel, Anika Söhner, Marc Maaß, Wolfgang Sauerwein, Dorothe Möllmann, Hideo Andreas Baba, Martin Kramer, Lutz Lüdemann

**Affiliations:** 1 Department of Radiotherapy, Medical Physics, University Hospital Essen, Essen, North Rhine-Westphalia, Germany; 2 Department of General and Visceral Surgery at Evangelical Hospital Wesel, Wesel, North Rhine-Westphalia, Germany; 3 Department of Pathology, University Hospital Essen, Essen, North Rhine-Westphalia, Germany; 4 Hospital of Veterinary Medicine, Department of Small Animal Surgery, Justus Liebig University Giessen, Giessen, Hesse, Germany; Universitatsklinikum Wurzburg, GERMANY

## Abstract

The purpose of this study was to assess the accuracy of fractional blood volume (*v*_*b*_) estimates in low-perfused and low-vascularized tissue using dynamic contrast-enhanced magnetic resonance imaging (DCE-MRI). The results of different MRI methods were compared with histology to evaluate the accuracy of these methods under clinical conditions. *v*_*b*_ was estimated by DCE-MRI using a 3D gradient echo sequence with k-space undersampling in five muscle groups in the hind leg of 9 female pigs. Two gadolinium-based contrast agents (CA) were used: a rapidly extravasating, extracellular, gadolinium-based, low-molecular-weight contrast agent (LMCA, gadoterate meglumine) and an extracellular, gadolinium-based, albumin-binding, slowly extravasating blood pool contrast agent (BPCA, gadofosveset trisodium). LMCA data were evaluated using the extended Tofts model (ETM) and the two-compartment exchange model (2CXM). The images acquired with administration of the BPCA were used to evaluate the accuracy of *v*_*b*_ estimation with a bolus deconvolution technique (BD) and a method we call equilibrium MRI (EqMRI). The latter calculates the ratio of the magnitude of the relaxation rate change in the tissue curve at an approximate equilibrium state to the height of the same area of the arterial input function (AIF). Immunohistochemical staining with isolectin was used to label endothelium. A light microscope was used to estimate the fractional vascular area by relating the vascular region to the total tissue region (immunohistochemical vessel staining, IHVS). In addition, the percentage fraction of vascular volume was determined by multiplying the microvascular density (MVD) with the average estimated capillary lumen, $\pi {\left(\frac{d}{2}\right)}^{2}$, where *d* = 8*μ*m is the assumed capillary diameter (microvascular density estimation, MVDE). Except for ETM values, highly significant correlations were found between most of the MRI methods investigated. In the cranial thigh, for example, the *v*_*b*_ medians (interquartile range, IQRs) of IHVS, MVDE, BD, EqMRI, 2CXM and ETM were *v*_*b*_ = 0.7(0.3)%, 1.1(0.4)%, 1.1(0.4)%, 1.4(0.3)%, 1.2(1.8)% and 0.1(0.2)%, respectively. Variances, expressed by the difference between third and first quartiles (IQR) were highest for the 2CXM for all muscle groups. High correlations between the values in four muscle groups—medial, cranial, lateral thigh and lower leg - estimated with MRI and histology were found between BD and EqMRI, MVDE and 2CXM and IHVS and ETM. Except for the ETM, no significant differences between the *v*_*b*_ medians of all MRI methods were revealed with the Wilcoxon rank sum test. The same holds for all muscle regions using the 2CXM and MVDE. Except for cranial thigh muscle, no significant difference was found between EqMRI and MVDE. And except for the cranial thigh and the lower leg muscle, there was also no significant difference between the *v*_*b*_ medians of BD and MVDE. Overall, there was good *v*_*b*_ agreement between histology and the BPCA MRI methods and the 2CXM LMCA approach with the exception of the ETM method. Although LMCA models have the advantage of providing excellent curve fits and can in principle determine more physiological parameters than BPCA methods, they yield more inaccurate results.

## Introduction

Capillaries form a microvascular network that supplies the tissue with nutrients. Changes in microcirculation can be used as a diagnostic indicator of abnormal lesions and to optimize the treatment of disease [1]. For example, the fractional tissue blood volume, *v*_*b*_, which is defined as the volume fraction inside the capillary bed [2], represents one of the most valuable parameters for noninvasive tumor grading and can be estimated using dynamic contrast-enhanced magnetic resonance imaging (DCE-MRI) [3, 4].

DCE-MRI can be performed with different MRI sequence settings and contrast agents, thus enabling detailed assessment of microvascular integrity [5, 6]. This MRI technique enables diagnostic estimation of individual tumor vessel parameters and assessment of the response of tumor vessels to various forms of therapy [7, 8]. Using intravascular contrast agents (also called blood pool contrast agents, BPCA), *v*_*b*_, and blood flow inside a voxel can be estimated. BPCA have the advantage that short acquisitions of 1-2 minutes are sufficient to determine vascular parameters. Imaging with well-established extravasating contrast agents (low-molecular contrast agents, LMCA) on the other hand require about ten minutes to acquire meaningful data [2].

DCE-MRI using LMCA enables determination of the interstitial volume fraction and permeability surface area product and other parameters. So far, the most widely used pharmacokinetic model applied to data of this kind in clinical routine is the extended Tofts model [9]. However, it has the disadvantage of not taking into account the dispersion of the contrast agent bolus while passing through the vascular system, which results in considerable underestimation of blood volume [10, 11]. Moreover, its parameter, *K*^trans^, combines various types of essential information on tissues structure and function into a single quantity.

Combined administration of an LMCA and a BPCA has the potential to separate more accurately the relevant physiological transport processes (perfusion and permeation) [2, 12, 13]. Improvement of MRI scanner hardware and image reconstruction software has led to an increase in image quality and temporal resolution combined with good spatial coverage, which makes an evaluation of more complicated models conceivable, e.g., the two-compartment exchange model (2CXM) [1, 2]. More elaborate models presumably describe the microvascular tissue structure and function more realistically and are likely to provide more detailed and reliable information on tumor pathophysiology.

Abnormal formation of new vessels by sprouting or splitting processes (neoangiogenesis), initiated by growth proteins secreted by the tumor for the sole purposes of its own nutrient supply, leads to an anarchistic vascular organization of largely dilated microvessels accompanied by significantly increased arteriovenous shunt flow [13–15]. The degree of angiogenesis is an essential biomarker providing information on the activity and malignancy of brain tumors [16]. Increasing density of tumor neovascularization is a prominent sign of increasing histological grade and progression of many types of tumors. Histologically, neovascularization is quantified by measurement of microvascular density [17].

Microvascular blood volume has become important in the study of tissue malignancy in low-perfused organs such as skeletal muscle and the breast [13, 18–20] as well as in highly perfused brain tumors [3, 4, 21–27]. Preclinical BPCA-DCE-MRI results suggest that tumor vascular permeability or tumor blood volume clearly correlates with tumor grade and tumor angiogenesis, as estimated by histological microvascular density quantification, and with tumor response to anti-angiogenic therapy [28]. In addition, the endothelial walls of tumor vessels become more permeable. Therefore, the simultaneous determination of endothelial permeability and tumor blood volume in a single test in the same subject is desirable.

In vivo studies of the well-described skeletal muscle of large mammals with DCE-MRI provide detailed insights into the contrast enhancement of low-perfused tissue, and thus provide a clearer understanding of the functional and structural composition of its tissue architecture. The findings from well-reproducible studies in healthy homogeneous tissue constitute an essential reference for the clinical detection and characterization of malignant tissue in humans. In particular, the pig is a well-suited biomedical non-rodent model for the pharmacokinetics of the human microvasculature because it shares various physiological similarities with humans, especially with regard to its cardiovascular system [29]. Pig hearts are about the same size as human hearts, and coronary blood flow, hemodynamics, and myocardial contractility are similar [30]. Moreover, using domestic pigs as a model allows choosing animals with a body weight similar to humans to ensure maximum comparability. Thus, in the present study, swine skeletal muscle was used to validate the ability of pharmacokinetic DCE-MRI models to quantify the blood volume fraction.

## Materials and Methods

### Animals

In the present study, 14 adult female pigs (German Landrace or hybrid form; age: approximately 20 weeks; body weight: 56 to 67 kg; no food restriction) were sacrificed. Before this study, another 6 pigs were used to develop and optimize the surgical technique [31] and to establish optimal DCE-MRI and anatomical MR imaging protocols. Two or three animals at a time were bought from a local pig breeding farm. Two weeks before the experiments they were housed together in a 20 square meter vivarium with species-appropriate entertainment facilities. Only healthy animals without known cardiovascular or musculoskeletal disorders were used. The pigs were not fed overnight prior to the experiment but had free access to water. The local institutional ethics committee, the Landesamt für Natur, Umwelt und Verbraucherschutz Nordrhein-Westfalen (Approval No. 84-02.04.2012.A208), authorized the animal study, and all experiments were performed in accordance with the German Animal Protection Act. An MRI-compatible monitoring device (Veris, Medrad, Germany) was used for monitoring heart rate and oxygen saturation. None of the pigs woke up during any phase of the experiments. At the end of the experiment, the pig was euthanized under a higher anesthetic dose and by the injection of T61 (0.3 mL/kg).

### Surgical Technique and Histology

All surgical procedures were performed, under aseptic conditions, in an operating room equipped for large animals. The pigs were premedicated via an intra- muscular injection of 30 mg/kg ketamine (ketamine 10%, Ceva Tiergesundheit GmbH, Germany), 2 mg/kg azaperone (Stresnil Janssen-Cilag GmbH, Germany), and 0.02–0.05 mg/kg atropine sulfate (Atropinsulfat, B. Braun Melsungen AG, Germany). A peripheral 20-G venous catheter was placed in a ear vein and, approximately 30 min after premedication, total intravenous anesthesia was initiated. A perfusor was used to inject 4-7 mg/kg/h propofol (Propofol-ratiopharm, Ratiopharm, Germany), 0.1-0.5 mg/kg/h midazolam (midazolam injection solution 0.5%, Germany), and 0.0015 mg/kg/h fentanyl (fentanyl citrate solution 3.9 mL/50 mL, Germany). A tracheal tube was placed (Hi-Contour cuffed tracheal tube, ID 8.0, Mallinckrodt, Ireland), and the pig was ventilated with a respiratory device (Fabius, Draeger, Germany). The tidal volume was set at 10 mL/kg, the respiratory rate at 12-14 breaths/min, and the positive end-expiratory pressure at 5 mbar. The tidal volume and frequency were adjusted to keep end-expiratory CO2 within 35-40 mmHg and to maintain at least 95% peripheral oxygen saturation. A central venous catheter (3-Lumen-ZVK-Set, ARROWgard Blue, Arrow, Germany) was placed in the jugular vein on the right side of the neck; the central venous catheter was used for administration of the contrast agent (CA).

In six experiments, MRI acquisitions were followed by obtaining muscle biopsies (1 × 1 × 1 cm) from the lateral (biceps femoris muscle), the cranial (rectus femoris muscle) and the medial (gracilis muscle and adductors) thigh muscles and from the lower leg muscle (gastrocnemius muscle). Except of one, these experiments were no subset of the MRI experiments. The tissue samples were fixed in formaldehyde (4.5%), embedded in paraffin and cut with a rotary microtome (HM355S, Microm International GmbH, Walldorf, Germany) into 2-3 *μ*m-thick sections.

Vascular endothelium was labeled using immunohistochemical staining with isolectin. The sections were deparaffinized in xylene and rehydrated in a descending alcohol series (100%, 95%, 70%) and tap water. Subsequently, the sections were placed in a 3% solution of H_2_O_2_ for 10 min and rinsed with wash buffer (DCS Innovative Diagnostik-Systeme Dr. Christian Sartori GmbH & Co. KG, Hamburg, Germany). This step was followed by a protein block with 10% donkey normal serum (DNS) in Dako Real Antibody Diluent (Dako Deutschland GmbH, Hamburg, Germany) for 60 min at room temperature. The sections were rinsed with wash buffer and stored at 4°C over night in a solution of isolectin (Biotinylated Bandeiraea simplicifolia lectin I Isolectin B4, Vector Laboratories, inc., Burlingame, USA) at a dilution of 1:100 or 1:500 with block buffer (10% DNS in Dako Real Antibody Diluent). After rinsing with wash buffer, the sections were overlayed for 45 min with streptavidin-HRP (Dianova GmbH, Hamburg, Germany) at a dilution of 1: 500 and then rinsed again. After that the sections were placed in diaminobenzidine (DAB) solution twice for five minutes and again rinsed with wash buffer. This was followed by counterstaining with hematoxylin and blueing under running tap water. The sections were dehydrated in ascending alcohol series (70%, 95%, 100%) and xylene and covered with Entellan (Merck, Darmstadt, Germany).

The samples were examined with a light microscope (Zeiss Axioplan, Carl Zeiss Microscopy GmbH, Jena, Germany). From each muscle section, 20 images (magnification 400×) were taken with a special camera. Images were taken randomly in a meandering manner, selecting only low-artifact fields of view, where possible without breaks or overlays. This was followed by semiautomatic analysis of the images with the microscope’s morphometry software (Zeiss Axio Vision, Axio Vs40 4.6.3.0, Jena, Germany).

#### Immunohistochemical Vessel Staining Estimation Method

**Fig 1:**
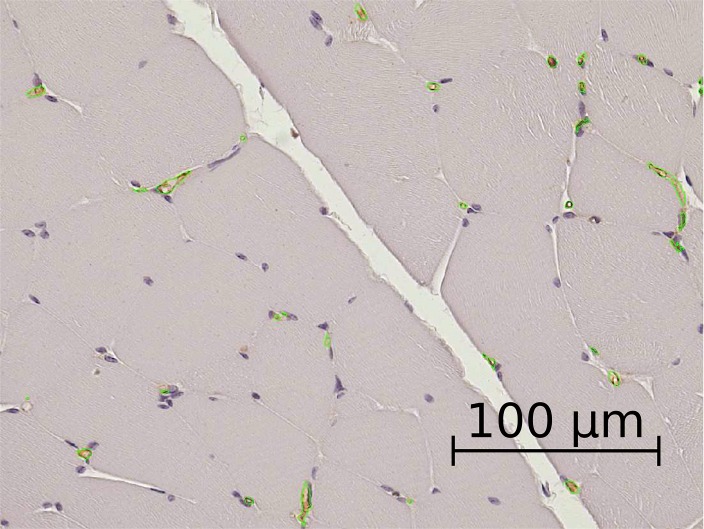
Light microscopy image of a histological preparation from the medial thigh muscle with vascular endothelium stained brown by isolectin. The selection was performed by semiautomatic analysis of the images with the microscope’s morphometry software to determine the area, number and diameter of the vessels. The outer borders of the segmented vessels are labeled in green.

The images of isolectin-stained sections were segmented using a first threshold set to label the whole tissue area including muscle cells, connective tissue, vessels, vessel lumina and other tissue structures. The tissue area was determined. A second threshold was set to label brown-colored endothelial cells (Fig 1). Manual correction was performed to include vessel lumina while excluding falsely detected areas such as cell nuclei. The area, number and diameter of the vessels were determined. S1 File

Parameter analysis was performed using Microsoft Excel 2010 (Microsoft, Washington, United States). First, the total tissue area was determined. The tissue areas composed of muscle cells, connective tissue, vessels and their lumina, and other tissue structures were determined in square microns using the morphometry software. Note that paraffin embedding leads to tissue shrinkage and makes intercellular spaces appear larger.

Thus, the fluid-filled portions of the intercellular spaces cannot be determined reliably. In a previous study, the percentage of interstitial fluid space (IFS) in frozen sections was determined [32]. We added this value to the morphometrically determined tissue area in this study to obtain the total tissue area. Then the percentage of the vascular area of the total tissue area was determined. The diameter of the vessels was calculated by the morphometry software in microns. Since tissue processing is associated with approx. 20% shrinkage [33], this shrinkage loss was added to the output value. In the following, we refer to this method of histological blood volume determination as immunohistochemical vessel staining (IHVS).

#### Microvascular Density Estimation Method

Using the sites of the vessels determined for IHVS, microvascular density (MVD) was calculated as the number of these vessels per square micrometer. For a second method to estimate the fractional blood volume histologically, the MVD was used to estimate the true vascular lumen. The MVD was multiplied by the average estimated capillary lumen, (*d*/2)^2^ ⋅ *π*, given by the mean capillary diameter, *d*, assumed to be *d* = 8*μ*m [34]. In the following, we refer to this method as microvascular density estimation (MVDE).

### MRI Technique

The pig was positioned supine on the scanner table. The MR scanner table had a built-in 32-channel coil (Siemens Magnetom Aera 1.5 T, Siemens Healthcare, Erlangen, Germany). A body surface coil (array body coil, 18 RF channels, Siemens Healthcare, Erlangen, Germany) was placed on the lower body of the pig. Heart rate and oxygen saturation were monitored with an MR-compatible device (Veris, Medrad, Germany).

Nine of the 14 pigs were investigated using MRI methods to estimate fractional blood volume. To create relaxation-rate-change-time curves, baseline magnetization and relaxation rates were determined before each dynamic acquisition using a T1w 3D gradient echo sequence (TWIST, Siemens Healthcare, Erlangen, Germany) with different flip angles (*α* = 5°, 10°, 20°, and 30°). The sequence parameters were: TR = 2.69 ms, TE = 0.86 ms, voxel size: 2.9 × 2.9 × 4.5mm^3^, 160 × 128 × 48 reconstruction matrix, frequency encoding in the axial direction, parallel imaging (GRAPPA) in 3D with 32 central k-space lines and an acceleration factor of 6. The central k-space region was 100%, i.e., k-space sharing was not used for the baseline acquisitions. To improve reproducibility of signal recordings, automatic sequence adjustments were turned off.

The subsequent first 100 dynamic acquisitions were performed using both, peripheral recording density and a central region of 20% each. The other settings were the same as for the baseline acquisitions, except flip angle *α* = 30°. k-space sharing ensured a temporal resolution of approx. 1.5 sec. The LMCA (Dotarem, Gadoteric Acid, 0.5 mmol/mL, molecular weight 0.56 kDa, Guerbet, France, 0.2 mL/kg body weight) was administered at an injection rate of 5 mL/sec during the dynamic scan starting after acquisition of the fifth image and was directly followed by a 20 mL saline flush injected at the same rate. The 100 3D GRE acquisitions with high temporal resolution were followed by 250 acquisitions with a read-out fraction of the central k-space of 50% and an identical peripheral k-space sampling density. These settings resulted in a lower temporal resolution (approx. 3.5 sec) for the wash-out of the LMCA.

After each acquisition with LMCA we performed a BPCA acquisition using the same sequence settings as before. After the fifth acquisition of the first dynamic sequence, 0.1 mL/kg body weight BPCA (0.25 mMol/mL gadofosveset trisodium, Vasovist Bayer Schering, Berlin, Germany/Ablavar, Lantheus Medical Imaging, Inc., USA) was injected via the central venous catheter at a flow rate of 5 mL/s followed by 20 mL saline using the same injection rate. The molecular weight of the active chelate is 957 Da, but bonding of the contrast agent to serum albumin effectively increases the molecular weight to 68 kDa, resulting in a compound with macromolecular properties [35, 36]. More details of the entire experimental setup can be found elsewhere [31, 32].

### Image Data Processing

The baseline 3D GRE images acquired with different flip angles were used to determine baseline longitudinal relaxation rate (*R*_10_) and magnetization (*M*_0_). Together with the k-space-shared dynamic acquisitions, the time-dependent 3D relaxation rate change maps Δ*R*_1_(*t*) were generated using the method of Li et al. [37]. Δ*R*_1_(*t*) was assumed to be proportional to the time-dependent contrast agent concentration. S2 File

To avoid partial volume effects, the estimated arterial input function (AIF), $c_{a}^{\left(est\right)}\left(t\right)$, was determined from voxels that definitely were completely in the abdominal aorta. The AIF volume of interest consisted of approx. 20 voxels with a total volume of approx. 750 mm^3^.

Morphological images and the pre- and post-LMCA difference images (T1-TSE) and the dynamic GRE images with local contrast agent administration were used for contouring the medial thigh muscle. For tracer kinetic modeling, averaged relaxation rate change time curves of these virtually segmented muscle regions were used.

We used the Amira Dev 5.2 visualization package (Mercury Computer Systems, Berlin, Germany) on a Debian Linux 64 bit workstation with eight 3.4 GHz processors and 16 GB RAM for virtual segmentation of the muscle area of interest and for calculation of the relaxation rate change time curves. The functionality of Amira 5.2 has been extended for the applicability of software packages that are included as link libraries in AmiraDev.

### Blood Volume Quantification Methods

Four different methods of blood volume quantification were applied—two using BPCA and two LMCA. They are briefly described in the following paragraphs. For detailed information we refer to the literature.

#### Extended Tofts Model (ETM)

The extended Tofts model is described in detail in [1, 2, 9]. It is mathematically described as follows:

$$\begin{array}{c} c_{t}\left(t\right)=v_{p}\cdot c_{a}\left(t\right)+K^{\text{trans}}\cdot e^{-tk_{\text{ep}}}⊗c_{a}\left(t\right), \end{array}$$ (1)

where *c*_*t*_(*t*) and *c*_*a*_(*t*) are the concentration-time curves of the tissue of interest and of the blood plasma of the artery supplying the tissue, respectively. *v*_*p*_, is the fractional plasma volume. In case of a compartimental description [38]:

$$\begin{array}{c} K^{\text{trans}}=\frac{F_{p}\cdot PS}{F_{p}+PS}. \end{array}$$ (2)

The permeability-surface product, *PS*, describes the exchange of the CA between the vascular and the interstitial compartment over the endothelial barrier. The transfer constant from the interstitial space back to the blood plasma, *k*_ep_, is defined as follows:

$$\begin{array}{c} k_{\text{ep}}=\frac{K^{\text{trans}}}{v_{e}}. \end{array}$$ (3)

Here, *v*_*e*_ is the interstitial volume fraction. Plasma perfusion *F*_*p*_ is related to blood perfusion *F* by the relationship *F*_*p*_ = *F* ⋅ (1 − Hct_*t*_), where Hct_*t*_ is the microvascular or tissue hematocrit level.

For LMCA data, we used the estimated arterial input function, $c_{a}^{\left(est\right)}\left(t\right)$, measured in the aorta, to calculate the arterial plasma concentration also known as arterial input function, *c*_*a*_(*t*), at the inlet of the vascular compartment of the tissue of interest, taking into account a time delay Δ*t*:

$$\begin{array}{c} c_{a}\left(t\right)=c_{a}^{\left(est\right)}\left(t-\Delta t\right). \end{array}$$ (4)

**Fig 2:**
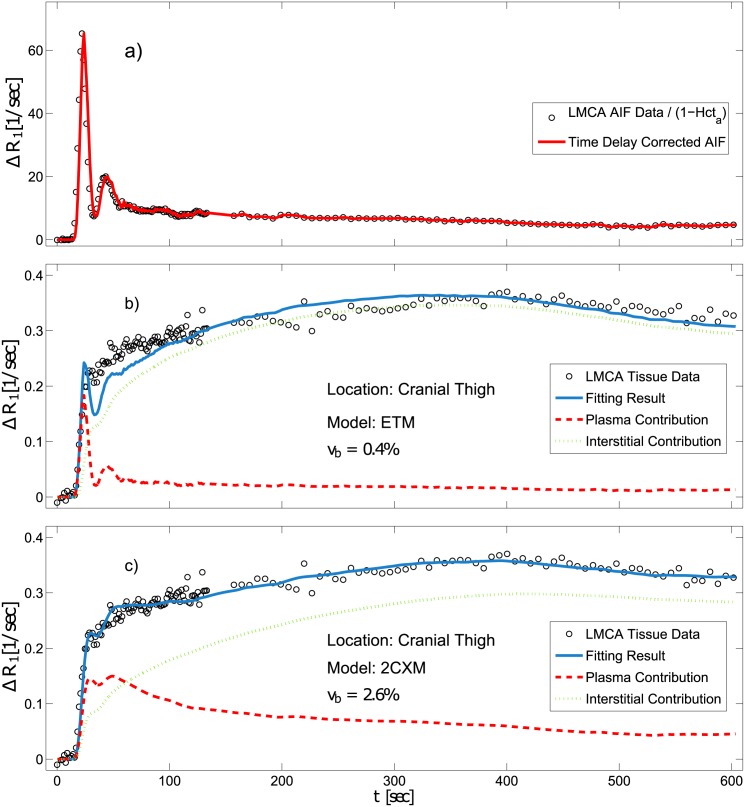
Representative LMCA relaxation-rate-change time curves measured a) in the aorta and b) and c) in the medial thigh muscle are shown using black circles. Fig a) shows the bolus-delay-corrected arterial input function (red solid line) related to the arterial hematocrit Hct_*a*_). Fig b) demonstrates the curve fitting result with the ETM and Fig c) the result with the 2CXM (red solid lines) and individual results for the plasma fraction (blue dot-dashed line) and the interstitial contribution (green dotted line). Obviously, the data are much better fitted using the 2CXM. Taking bolus broadening into account, the 2CXM yields a significantly larger area under the curve for the plasma contribution compared to the ETM, where the plasma compartment is merely the AIF scaled down with the plasma volume. As a result of the larger blood plasma fraction, the interstitial contribution using the 2CXM is lower, especially during the CA uptake period.

Fig 2a) shows an example of the corrected AIF measured in the abdominal aorta with use of LMCA. The arterial blood concentration, *c*_*a*,*b*_(*t*), is directly related to the arterial plasma concentration, *c*_*a*_(*t*), by the arterial hematocrit level: *c*_*a*,*b*_(*t*) = *c*_*a*_ ⋅ (1 − Hct_*a*_) ([2]). For the arterial blood of the pig, we used an estimated value of Hct_*a*_ = 0.4 ([39, 40]). The concentration-time curve in the tissue of interest is given by *c*_*t*_(*t*), and the fractional plasma volume, *v*_*p*_, is related to the fractional blood volume by the relationship *v*_*p*_ = *v*_*b*_ ⋅ (1 − Hct_*t*_). In this study, we assumed the level of microvascular or tissue hematocrit, Hct_*t*_ = 0.2, to be 50% of the arterial hematocrit [41].

Fig 2b) shows Δ*R*(*t*) of the fit of the medial thigh and the fit of ETM to Δ*R*(*t*) data. Also shown are the blood plasma (blue dashed line) and the interstitial contribution (green dots). The ETMs vascular contribution is the AIF downscaled by the factor *v*_*p*_.

#### Two-Compartment Exchange Model (2CXM)

A more general model for the distribution of contrast agent in tissue is the two-compartment exchange model [1, 2]. Unlike the ETM, the 2CXM accounts for broadening of the AIF in the vascular compartment and can be described by the following two mass balance equations:

$$\begin{array}{c} v_{p}\frac{\text{d}c_{p}\left(t\right)}{\text{d}t}=F_{p}\cdot c_{a}\left(t\right)-F_{p}\cdot c_{p}\left(t\right)+PS\cdot c_{e}\left(t\right)-PS\cdot c_{p}\left(t\right) \end{array}$$ (5)

$$\begin{array}{c} v_{e}\frac{\text{d}c_{e}\left(t\right)}{\text{d}t}=PS\cdot \left(c_{p}\left(t\right)-c_{e}\left(t\right)\right). \end{array}$$ (6)

The total tissue concentration is calculated as

$$\begin{array}{c} c_{t}\left(t\right)=v_{p}\cdot c_{p}\left(t\right)+v_{e}\cdot c_{e}\left(t\right). \end{array}$$ (7)

Here, *c*_*e*_ is the interstitial CA concentration. Fig 2c) shows Δ*R*(*t*)_1_ measured using LMCA and the fit of the 2CXM in comparison to ETM to the same data.

#### Bolus Deconvolution (BD)

To describe the distribution of the BPCA in the vascular system of the tissue according to the indicator dilution theory, we estimated the tissue blood concentration, *c*_*b*_, by convolution of the first 45 seconds of the AIF with a single exponential [42]:

$$\begin{array}{c} c_{b}\left(t\right)=\xi \cdot F\cdot c_{a}\left(t\right)⊗H\left(t\right), \end{array}$$ (8)

where *F* is blood perfusion and *H*(*t*) the residue function with the vascular mean transit time, *MTT*:

$$\begin{array}{c} H\left(t\right)=e^{-t/MTT}, \end{array}$$ (9)

and the blood volume is calculated via the central volume theorem [42]:

$$\begin{array}{c} v_{b}=MTT\cdot F. \end{array}$$ (10)

Factor *ξ* in Eq (8) is a proportionality constant which depends on the difference in hematocrit levels between capillaries and large vessels. It compensates for the fact that only the plasma volume is accessible to the contrast agent. Technically, it also depends on the density of the tissue, which is set to 1.0 g/mL in this study. In this way we can assume:

$$\begin{array}{c} \xi =\frac{1-{\text{Hct}}_{t}}{1-{\text{Hct}}_{a}}. \end{array}$$ (11)

**Fig 3:**
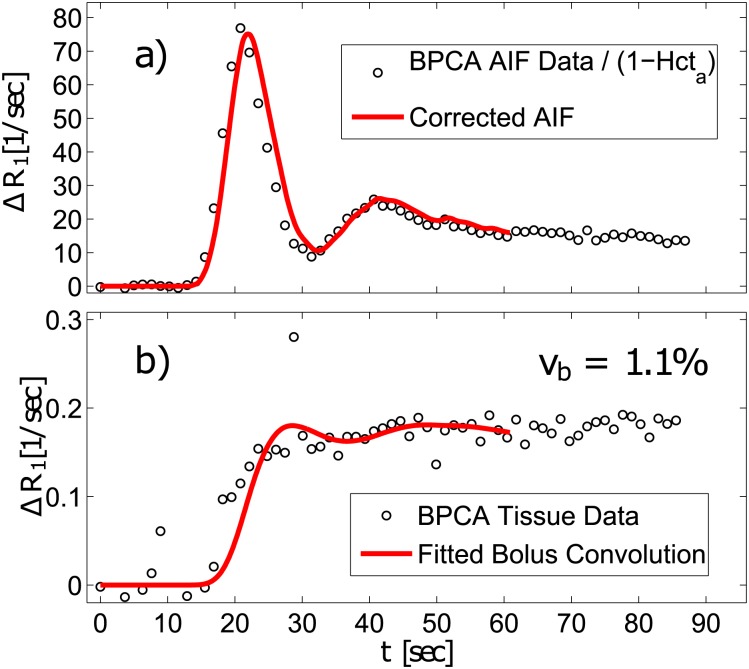
BPCA data recorded from CA-induced time-dependent relaxation rate changes in a) the aorta (related to the arterial hematocrit level Hct_*a*_)) and b) the medial thigh muscle (black circles) of the same experiment as the examples in Fig 2. Additionally, in a) the time delay and bolus-dispersion-corrected AIF and in b) one example of the fitting results with the BD method (red solid lines) is shown. As indicated by the statistical results (Table 1), *v*_*b*_ is determined lower by BD compared with 2CXM (Fig 2) and EqMRI (Fig 4).

To calculate the AIF *c*_*a*_(*t*) for the capillary compartment we corrected $c_{a}^{\left(est\right)}\left(t\right)$ for delay Δ*t* and dispersion times 1/*β*:

$$\begin{array}{c} c_{a}\left(t\right)=c_{a}^{\left(est\right)}\left(t+\Delta t\right)⊗h\left(t\right), \end{array}$$ (12)

where

$$\begin{array}{c} h\left(t\right)=\beta \cdot e^{-\beta t} \end{array}$$ (13)

is the vascular transport function that describes bolus dispersion during effective transit time 1/*β* from the site of AIF measurement to the entry of the particular region of interest. Details of this method can be found for example in [43–46]. Fig 3a presents relaxation rate changes, Δ*R*_1_(*t*), of the measured and dispersion-corrected AIF. Fig 3b presents an example of the BD fit to the blood tissue Δ*R*_1_(*t*) curve measured in the medial thigh muscles of intravascular contrast agent enhancement in the tissue after bolus injection.

#### Equilibrium MRI (EqMRI)

**Fig 4:**
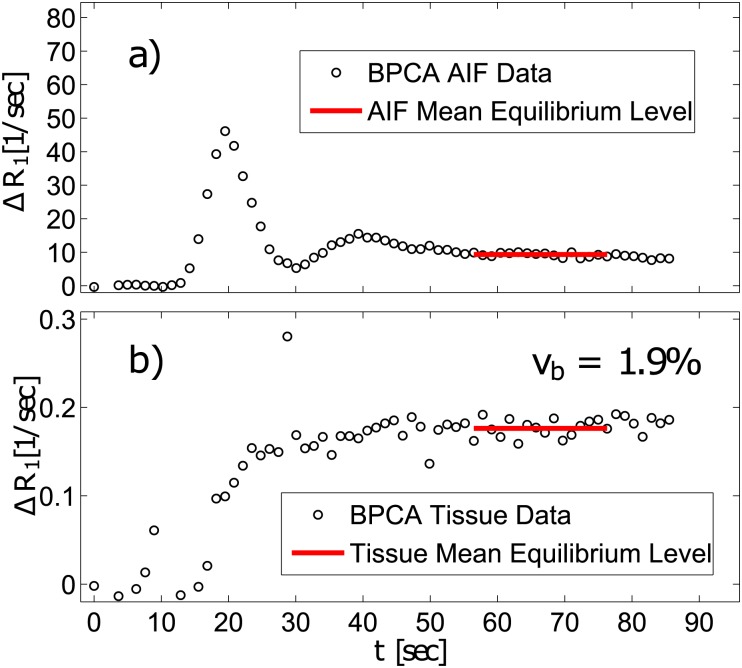
For the EqMRI method, the same BPCA relaxation rate changes as in Fig 3 are shown as black circles. No correction with respect to the arterial hematocrit level was performed at this point. Red solid lines represent the estimated average equilibrium states of a) the AIF in the aorta and in b) the medial thigh muscle (black circles) according to Eq (14) are shown.

Starting 40 seconds after bolus arrival, both the concentration-time curves of the BPCA-AIF and the curve of the tissue blood were averaged over a period of 20 seconds. In this period, we assumed an approximate equilibrium of contrast agent concentration. To estimate the blood volume, these two averages were related to each other [47]:

$$\begin{array}{c} v_{b}=\xi^{-1}\cdot {\frac{\left\langle c_{b}\left(t\right)\right\rangle }{\left\langle c_{a}\left(t\right)\right\rangle }|}_{t=40s}^{t=60s}\;\;, \end{array}$$ (14)

with *ξ* as defined in Eq (11). Fig 4 shows the method based on the same data as in Fig 3 for the AIF (a) and the tissue blood signal (b). Here, the red solid lines represent the mean values of the relaxivity rate change over an interval of 20 sec, starting 40 sec after bolus arrival.

### Curve Fitting

To produce an approximation of the relaxation rate change time curve information that would have been obtained by sampling the signal at a higher rate, we performed temporal upsampling to 0.1 sec by linear interpolation. This procedure was carried out to propagate the model fit flexibility by a higher possible temporal interframe correspondence between the estimated AIF, $c_{a}^{\left(est\right)}$, and the tissue time course, *c*_*t*_(*t*). For both model fits, ETM and 2CXM, we used the first ten min of the relaxation-rate-change time curves.

We implemented the curve fitting algorithm in MATLAB 2011b (The MathWorks, Natick, Massachusetts, USA) and applied MATLAB’s constrained nonlinear optimization algorithm (fmincon) with a sequential quadratic programming method. A multi-start method with 1,000 starting points was used to find the global optimal solution for the set of model parameters. The boundaries of the parameter space were set to a wide range and were set the same for all models. The starting value of the time delay, Δ*t*, was estimated from the individual bolus arrival time (BAT).

### Statistical Analysis

Statistical analysis was performed using Microsoft Excel 2010 (Microsoft, Redmond, Washington, USA) and MATLAB R2011b. All results were expressed as medians with interquartile ranges, IQR (the difference between third and first quartile), as variances. Correlations were calculated using Pearson (*r*_*P*_) and Spearman (*r*_*S*_) correlation coefficients considering *p* < 0.05 to indicate significant differences.

## Results

**Fig 5:**
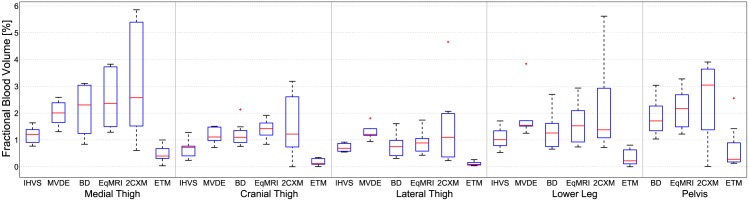
Box plots of the model parameter *v*_*b*_ obtained with the different models and methods in the different skeletal muscle regions of the pigs’ hind legs.

Isolectin staining of the medial thigh muscle labeling endothelium enabled determination of the vascular area (Fig 1). A clear separation of the different compartments is apparent. However, owing to collapse of blood vessels due to the disappearance of blood pressure, this method most likely underestimates the in vivo blood volume. The percentage of the segmented vascular area relative to the total muscle area was compared to the in vivo blood volume determined by MRI, see Table 1. Table 1 and Fig 5 present the *v*_*b*_ values obtained with the two histological (IHVS and MVDE) and with four different MRI techniques (BD, EqMRI, ETM, and 2CXM) in different muscle groups [46] of the pigs’ hind legs.

**Table 1:** Median values of the model parameter *v*_*b*_ with first (Q1) and third quartiles (Q3) and interquartile ranges (IQR) obtained with the different models and methods in the different skeletal muscle regions of the pigs’ hind legs. Also shown are the mean values of the contrast-to-noise ratio (CNR) of the CA time courses for each muscle region. The mean CNRs of the AIFs were 80 ± 40 (92 ± 37) for the BPCA (LMCA) measurements, respectively.

Tissue	Technique	CNR	Method	Median [%]	Q1 [%]	Q3 [%]	IQR [%]
Med. Thigh	Histology		IHVS	1.2	1.0	1.4	0.4
			MVDE	2.0	1.7	2.4	0.7
	MRI	29 ± 15	BD	2.3	1.3	3.0	1.7
			EqMRI	2.4	1.6	3.7	2.2
		42 ± 16	2CXM	2.6	1.6	5.4	3.8
			ETM	0.4	0.4	0.7	0.3
Cran. Thigh	Histology		IHVS	0.7	0.5	0.8	0.3
			MVDE	1.1	1.0	1.4	0.4
	MRI	22 ± 9	BD	1.1	1.0	1.3	0.4
			EqMRI	1.4	1.3	1.6	0.3
		30 ± 11	2CXM	1.2	0.8	2.6	1.8
			ETM	0.1	0.1	0.3	0.2
Lat. Thigh	Histology		IHVS	0.7	0.6	0.8	0.3
			MVDE	1.2	1.2	1.4	0.2
	MRI	17 ± 5	BD	0.7	0.4	0.9	0.5
			EqMRI	0.9	0.6	1.0	0.4
		32 ± 19	2CXM	1.1	0.4	2.0	1.6
			ETM	0.1	0.1	0.1	0.1
Lower Leg	Histology		IHVS	1.0	0.8	1.3	0.5
			MVDE	1.5	1.5	1.7	0.2
	MRI	20 ± 11	BD	1.3	0.8	1.6	0.8
			EqMRI	1.5	0.9	2.0	1.1
		32 ± 15	2CXM	1.4	1.1	2.8	1.7
			ETM	0.2	0.1	0.6	0.5
Pelvis	MRI	12 ± 5	BD	1.7	1.4	2.3	0.9
			EqMRI	2.2	1.5	2.7	1.1
		20 ± 9	2CXM	3.1	1.8	3.6	1.8
			ETM	0.3	0.2	0.7	0.5

For the respective muscle areas, the different methods yield comparable *v*_*b*_ values. In particular, there is good agreement between MRI and histological MVDE. One exception are the ETM results, which are in a range of only about 10-20% of the results obtained with the different MRI methods investigated. Although the 2CXM coincides in median and mean values with those of the two BPCA methods and MVDE, it yields high variances. Overall, the highest median values were found for the medial thigh muscles. The lowest values were obtained for the cranial and lateral thigh muscles.

In the medial thigh muscle, histological measurements yielded a median $v_{b}^{MVDE}of$ 2% and a median $v_{b}^{IHVS}of$ 1.2%. The three methods EqMRI, BD and 2CXM yielded higher volumes of 2.3%, 2.4% and 2.6%, respectively. The medians of MVDE (IHVS) were found to be at lower levels of 1.1% (0.7%) to 1.5% (1.0%) in the cranial and lateral thigh muscles and in the lower leg. For all muscle areas, much lower median values were obtained with the ETM, ranging from 0.1% (cranial and lateral thigh) to 0.4% (medial thigh). Histology yielded the lowest variance, expressed by the interquartile range (IQR), in all muscle areas. In the medial thigh, for example, MVDE (IHVS) IQR was 0.7% (0.4%) versus 1.7%, 2.2% and 3.8% for BD, EqMRI and 2CXM, respectively. In the other muscle groups, only the intravascular methods, BD and EqMRI, yielded IQRs that were partially as low as those of histology (i.e., in the cranial thigh).

Each of the four median value pairs, measured in the muscle areas of medial, cranial, lateral thigh and lower leg, were correlated for the different measuring methods. Significantly high Pearson correlation coefficients were only found for BD and EqMRI (*r*_*P*_ = 0.99, *p*_*P*_ < 0.01), for MVDE and 2CXM (*r*_*P*_ = 0.99, *p*_*P*_ < 0.01), and for IHVS and ETM (*r*_*P*_ = 0.96, *p*_*P*_ < 0.05). Table 2 presents the Spearman correlation coefficients for the individual values of the 9 MRI experiments for the different muscle areas and for the comparisons of MRI methods. In medial thigh muscle, significant correlations between all methods were observed. Correlation was highest between the two BPCA methods, EqMRI and BD (*r*_*S*_ = 0.95, *p*_*S*_ < 0.001), and lowest between BD and ETM (*r*_*S*_ = 0.75, *p*_*S*_ < 0.05). Except for the pelvis, the correlation between EqMRI and BD was very high and clearly significant for all muscle segments, with values up to *r*_*S*_ = 1.0. In all other muscle regions, no significant correlation was detected between the ETM and the other MRI methods. An exception was the measurement in the pelvis, where a correlation between the ETM and the 2CXM and between the ETM and EqMRI of both *r*_*S*_ = 0.77 (*p*_*S*_ < 0.05) was maintained. Between the intravascular contrast agent methods and the 2CXM, significant correlations were revealed for all muscle regions, with values of *r*_*S*_ = 0.78 (*p*_*S*_ < 0.05) to *r*_*S*_ = 0.89 (*p*_*S*_ < 0.01). The correlation between the combined 5 × 12 values of all muscle areas in all experiments yielded significant correlations between all MRI methods with values of *r*_*S*_ = 0.47 (*p*_*S*_ < 0.01) between the ETM and the 2CXM and *r*_*S*_ = 0.94 (*p*_*S*_ < 10^−20^) between BD and EqMRI.

**Table 2:** Spearman correlation coefficients of the individual results of the four MRI methods for the muscle groups in the hind legs of the pigs and Wicoxon rank sum test results between the different MRI methods and between MRI and histology.

		EqMRI-BD	BD-ETM	BD-2CXM	ETM-2CXM	2CXM-EqMRI	ETM-EqMRI	BD-MVDE	EqMRI-MVDE	ETM-MVDE	2CXM-MVDE
Med. Thigh	*r*_*S*_ (*p*_*S*_)	0.95 (<10^−3^)	0.75 (<0.05)	0.88 (<10^−2^)	0.88 (<10^−2^)	0.88 (<10^−2^)	0.83 (<10^−2^)	–	–	–	–
	*p*_*W*_	n.s.	<10^−4^	n.s.	<10^−3^	n.s.	<10^−4^	n.s.	n.s.	<0.05	n.s.
Cran. Thigh	*r*_*S*_ (*p*_*S*_)	1.0 (<10^−5^)	0.28(n.s.)	0.78 (<0.05)	0.58 (n.s.)	0.78 (<0.05)	0.28 (n.s.)	–	–	–	–
	*p*_*W*_	n.s.	<10^−4^	n.s.	<10^−3^	n.s.	<10^−4^	<0.05	<0.05	<0.05	n.s.
Lat. Thigh	*r*_*S*_ (*p*_*S*_)	1.0 (<10^−4^)	−0.27 (n.s.)	0.89 (<10^−2^)	−0.3 (n.s.)	0.89 (<10^−2^)	−0.23 (n.s.)	–	–	–	–
	*p*_*W*_	n.s.	<10^−4^	n.s.	<0.01	n.s.	<10^−4^	n.s.	n.s.	<0.05	n.s.
Lower Leg	*r*_*S*_ (*p*_*S*_)	0.99 (<10^−4^)	0.29 (n.s.)	0.89 (<10^−2^)	0.27 (n.s.)	0.88 (<10^−2^)	0.28 (n.s.)	–	–	–	–
	*p*_*W*_	n.s.	<0.01	n.s.	<10^−3^	n.s.	<10^−4^	<0.05	n.s.	<0.05	n.s.
Pelvis	*r*_*S*_ (*p*_*S*_)	0.58 (n.s.)	0.52 (n.s.)	0.82 (<0.05)	0.77 (<0.05)	0.78 (<0.05)	0.77 (<0.05)	–	–	–	–
	*p*_*W*_	n.s.	<0.05	n.s.	n.s.	n.s.	<0.01	–	–	–	–
Combined	*r*_*S*_ (*p*_*S*_)	0.94 (<10^−20^)	0.54 (<10^−3^)	0.81 (<10^−10^)	0.47 (<10^−2^)	0.79 (<10^−10^)	0.63 (<10^−5^)	–	–	–	–
	*p*_*W*_	n.s.	<10^−11^	n.s.	<10^−9^	n.s.	<10^−12^	<10^−2^	n.s.	<10^−6^	n.s.

We used the Wilcoxon rank sum test to identify significant differences between the median values for *v*_*b*_ of different MRI techniques (*p*_*W*_ < 0.05 indicates that the test rejects the null hypothesis of equal medians at the 5% significance level). For the comparison of ETM results with the results of the other methods, no significant correspondence between the medians was detected with one exception: there was a match between the ETM and 2CXM in the pelvis. The last four columns in Table 2 show the Wilcoxon signed rank test p-values for determination of significant matches between the median *v*_*b*_ results of MVDE histology and all MRI methods. Comparison of histological medians with those of the 2CXM yielded no significant differences for any of the muscle areas investigated. For the ETM, however, the medians were significantly different from those of histology for all muscle groups. Significant differences were found in the cranial thigh muscles for EqMRI and both in the cranial thigh muscles and in the lower log for BD.

## Discussion

In the present study, we found good agreement between the results obtained with MRI and histology and between the results obtained using the different methods and models with the exception of IHVS histology and the ETM. For all techniques and methods, consistently different *v*_*b*_ values were found in the different muscle areas investigated. The ETM yielded unrealistic low blood volumes of less than one percent.

Very good correlations between the median blood volumes in all experiments with good conformity of absolute values in a plausible range and with low variance were obtained for the two BPCA methods, BD and EqMRI. Comparable *v*_*b*_ medians were obtained with MVDE histology, BD, EqMRI, and the 2CXM in the different muscle groups. Significant correlations were found for the median values measured in the four different muscle groups with MVDE (IHVS) histology and the 2CXM (ETM).

The MRI methods, however, yielded much larger *v*_*b*_ IQRs than histology, and the differences were especially pronounced for use of the 2CXM.

Table 3 lists average blood volume values reported in the literature for different skeletal muscles in different regions and obtained with different methods of analysis. For data obtained by MRI, the contrast agents and imaging techniques used by the investigators are provided. Because some authors do not report the volume of blood but the plasma volume as a result, we calculated the missing volume for all studies using the assumptions mentioned in the present article (*v*_*p*_ = *v*_*b*_(1 − Hct_*t*_), where Hct_*t*_ = 0.2). Reported average *v*_*b*_ values range from 1.5% (measured by MRI in the posterior hind limb) to approx. 5% (measured by MRI in the pectoral muscle and neck of humans). Our results are consistent with the literature in that different muscle regions have different blood volumes and in that the volumes measured are also roughly comparable. In other muscle regions, e.g., in [48], *v*_*b*_ = 2.5% was obtained in the anterior leg using the Patlak model and an intermediate contrast agent (Gadomer), which roughly corresponds to the medial thigh muscle in our study, where we found *v*_*b*_ = 2.6% using the 2CXM, and in the dorsal hind leg *v*_*b*_ = 1.5%, which corresponds to the lateral and cranial thigh muscle in our study with *v*_*b*_ = 1.1 or 1.2% with the 2CXM. As in the studies of Ruotsalainen and Raitakari [49, 50], we found higher *v*_*b*_ values in the anterior femoral region (medial thigh muscles). The higher *v*_*b*_ values in the anterior muscle area near the femoral artery (both in our study and those of others and for MRI and histology) might be attributable to the higher proportion of larger vessels in this area.

**Table 3:** Comparison of blood and plasma volume fractions of skeletal muscle of different species reported in the literature.

Species	Skeletal Muscle Location	*v*_*b*_ [%]	*v*_*p*_ [%]	Method	Contrast Agent/Tracer	Ref.
Rabbit	Anterior Leg	2.5 ± 0.7	(2.0 ± 0.6)^a^	SPGR, *T*_1_, Patlak model	Gadomer (17-35 kDa)	[48]
	Dorsal Leg	1.5 ± 0.4	(1.2 ± 0.3)^a^			
Rabbit	Soleus Muscle	(4.3 ± 1.0)^a^	3.4 ± 0.8	TAPIR, Δ*R*_1_, Ext. Tofts model	Gadomer (17-35 kDa)	[51]
	Tibialis Anterior	(2.6 ± 0.9)^a^	2.1 ± 0.7			
Human	Pectoral	(5.0 ± 1.3)^a^	4.0 ± 1.0	SRTF, *T*_1_, 2CXM	Gd-DTPA (0.94 kDa)	[13]
Human	Neck	4.9 ± 1.3	(3.9 ± 1.0)^a^	DCE-CT, 2CXM	Iopromide	[52]
Human	Femoral Region	3.3 ± 0.1	(2.6 ± 0.1)^a^	PET	[^15^O]CO	[50]
Human	Femoral Posterior	3.3 ± 0.7	(2.6 ± 0.6)^a^	PET	[^15^O]CO	[49]
	Femoral Anterolateral	4.7 ± 0.6	(3.8 ± 0.5)^a^			
Rabbit	Leg	3.1 ± 0.3	(2.5 ± 0.2)^a^	PET	[^15^O]CO	[53]
Rabbit	Thigh	1.8 ± 0.1	(1.4 ± 0.1)^a^	PET	[^15^O]CO	[54]
Domestic Pig (50 kg)	Abdomen	(2.8 ± 0.5)^a^	2.2 ± 0.4	Nuclear Hematology	^125^Iodine	[55]
Piglets (5 kg)	(not specified)	3.6 ± 0.9	2.9 ± 0.7	Nuclear Hematology	^99*m*^Tc, ^51^Cr	[56]
Guinea Pig (0.4-0.9 kg)	Soleus	2.1 ± 0.4	(1.7 ± 0.3)^a^	Stereology	-	[57]

^*a*^ Assuming Hct_*t*_ = 0.2.

Published blood or plasma volumes determined with different techniques and methods in different skeletal muscle areas, comp. Table 2.

An element of uncertainty in the determination of *v*_*p*_ is already introduced with the choice of systemic or arterial hematocrit. Systemic hematocrit is typically determined in venous blood. However, in pharmacokinetic modeling, i.e., in Eq (14), the arterial hematocrit is required, which is slightly lower than venous Hct. True arterial hematocrit levels are unknown, particularly in clinical trials of humans. Moreover, although the contrast agents only enter the plasma volume, some studies did not take into account the difference of tissue hematocrit and systemic hematocrit. The impact of the ratio of tissue to arterial hematocrit is described in Eq (11) for the bolus deconvolution method and in Eq (14) for the equilibrium method. Using the same values for both parameters would yield approx. 30% lower blood volumes. Measurement of individual arterial hematocrit would be useful since individual hematocrit levels may differ even among animals of the same sex, weight and breed and thus affect MRI blood volume estimates [40]. Reference [40] (p. 44, table below, row “Deutsche Landrasse”) reports a standard deviation for Hct_*a*_ levels measured in 50 individuals of 0.03, mean(±2SD) of 0.36(±0.06) and minimal (maximum) Hct_*a*_ of 0.29 (0.44). To estimate the influence of the lack of knowlege about individual Hct_*a*_ levels on the variability of our *v*_*b*_ results, we assume microvascular Hct to be independent of arterial Hct and exemplarily calculate median *v*_*b*_ values estimated with EqMRI to 2.3% for Hct_*a*_ = 0.42 and 2.8% for Hct_*a*_ = 0.3 instead of *v*_*b*_ = 2.4 with Hct_*a*_ = 0.4 (Table 1). However, omission of this measurement results in a small estimated difference of 0.5% compared to the IQRs shown in Table 1 (2.2% in the considered exemplarily case).

Hct_*a*_ was chosen based on the veterinary literature for pigs of similar breed and weight [39, 40]. Since intercapillary hematocrit can be as low as 20-40% of the systemic hematocrit (see [41] and refrences therein), intercapillary hematocrit has a stronger influence than tissue hematocrit in determining capillary blood volume. Exact determination of capillary blood volume using DCE-MRI would require exclusion of small vessels such as venules and arterioles. In the present study, all vessels were included in the analysis because current technology does not detect noncapillary vessels of sub-voxel size. On the other hand, maximum comparability between experiments was aimed at. An average vessel diameter of 8 *μ*m for MVDE was used in the present study. Reports in the literature describe capillary diameters between 5 and 10 *μ*.

Equating arterial and tissue hematocrit in Eqs (1) and (5) of the two-compartment model mass equations for the two LMCA methods leads to an underestimation of *v*_*b*_. For clinical application of tracer kinetic methods, the problem of inserting the correct hematocrit into the equations for each voxel is even more difficult, as hematocrit in tumorous tissue is highly inhomogeneous and might differ markedly from levels in healthy tissue.

Gadofosveset trisodium was used as intravascular contrast agent for blood volume determination based on the indicator dilution theory. It is a clinically approved gadolinium-based MRI contrast agent used as a blood pool contrast agent due to its reversible binding to endogenous human serum albumin and its high longitudinal relaxivity at low magnetic field strengths [35]. At low concentrations, 90% of the agent binds to human serum [58]. Thus, most of the agent remains within the vascular space for the first minute after administration, with only a small proportion of up to 10% undergoing extravasation. However, in pigs, binding of this contrast agent to albumin is slightly lower, 83-87%, but still high compared with other animals [59]. Gadofosveset binding to albumin leads to a marked increase in the relaxation rate [35]. Therefore, extravasation of unbound gadofosveset is negligible in the determination of physiological parameters using BPCA methods in healthy tissue.

However, tumor vasculature is impaired and the endothelial wall of tumor vessels are more permeable. Although our study is motivated by tumors, the results found in healthy muscle tissue cannot be directely transferred to estimations of these parameters in tumor tissue.

Donahue et al. described the relationship between vascular proton exchange rates and the accuracy and precision of tissue blood volume estimates using intravascular T1 contrast agents [60]. To minimize blood volume underestimation in fast exchange models they propose to use spoiled gradient echo pulse sequences with short TRs, which should be much smaller than the intravascular postcontrast T1. Moreover, the flip angle should not be too small. Since in our experiments T1 was on the order of 1500 ms, *α* = 30° and TR = 2.7 ms, artifacts due to capillary-interstitial proton exchange are minimal. However, the high contrast concentration during first pass of nonextravasating intravascular CA may result in a state closer to the slow proton exchange regimen and thus lead to a flattened bolus and *v*_*b*_ underestimation. These theoretical predictions are in good agreement with our experimental findings. The median values obtained with BD were on average 20% below those provided by EqMRI. Another factor contributing to these results, however, might be an overcorrection for dispersion in the BD case.

Schwarzbauer et al. proposed a steady-state intravascular CA quantification method of blood volume, which basically corresponds to the method termed EqMRI in our study [47]. For blood volume determination in the myocardium of rats overestimation resulting from T1 differences between blood and extravascular space, due to exclusive proton magnetization exchange between these spaces, was taken into account [61, 62]. The resulting perfusion dependence of T1 images acquired with a 2D FLASH sequence was used to correct regional blood volume. For low perfused tissues as investigated in the present study, the perfusion dependence of magnetization alteration can be neglected because, due to the long mean transit times, magnetization is already at the arterial end of the capillaries in steady state.

Because the site where the AIF is measured is close to the edge of the field of view, degradation of measurement accuracy through inflow effects cannot be fully excluded. For AIF extraction in the aorta using a 3D spoiled gradient echo DCE-MRI sequence with frequency encoding gradient in axial direction, Roberts et al. recommend an optimal distance of the extraction location of the AIF from the edge of the outermost axial scan of at least 20-30 mm to avoid underestimation of blood volume due to blood inflow [63]. The distance of the FOV edge from the nearest voxels of the selected AIF region in our study averaged 17 ± 8 mm and the selected voxels stretched over an average area of 39 ± 12 mm inside the FOV. No significant systematic relationship was found between the *v*_*b*_ results and the corresponding distances, expansion in the FOV of the AIF voxel areas or the quotient of both. However, we did not correct our data for B1 field inhomogeneities, which may have influenced both the median MRI *v*_*b*_ value and the uncertainties.

There were at least 30 minutes between the MRI examinations with the two contrast agents, LMCA and BPCA. Because gadoteric acid has a half-life of approx. 1.5 hours, we may assume that the effect of ongoing degradation of gadoteric acid during the 50-second BPCA measurement is negligible.

The ETM disregards bolus dispersion of the vascular contribution, leading to significant underestimates of the blood volume. On the other hand, the 2CXM has 5 model parameters including the time delay and may therefore be prone to overfitting. This would explain the large variance of *v*_*b*_ obtained with this model. The BD additionally takes into account bolus dispersion, making it theoretically more prone to overestimations of *v*_*b*_ and to a greater variance of results. This expectation, however, was not confirmed by our results.

The duration of the image acquisition period for LMCA models influences the results of curve fitting [64, 65]. Analyzing the results of all the model parameters for different acquisition times of both, the blood and the interstitial volume, we selected an acquisition period of 10 min. This acquisition duration (AD) provided the best fit for the 2CXM and the optimal match between the functional parameters of ETM and 2CXM. For example, in medial thigh muscle, the blood volume increased by 6% at an AD of 10 min relative to the value at AD = 6 min when using the 2CXM. The IQR, however, was reduced by 16%. On the other hand, for the ETM, the relative change in blood volume between these acquisition durations was 40% for the median and minus 5% for the IQR.

Luypaert et al. emphasize the importance of the acquisition period as well as the temporal resolution and the contrast-to-noise ratio (CNR) for the accuracy of model parameter estimation with the 2CXM [64]. They define the CNR as the ratio of the maximum tissue concentration to the standard deviation of the noise. They conclude that in order to keep the relative errors in determining the four model parameters using the 2CXM within a range of ±20%, demands must be made that cannot be met by currently available technology. However, their recommendations regarding temporal resolution (5 sec) and AD (110 sec) are met by our experimental setup. In contrast, the proposed requirement of a CNR of 40 is not met by all experiments in our study, since our LMCA CNR is in the range of 20 ± 9 (pelvis) to 42 ± 16 (medial thigh), which might be an additional explanation for the larg variations in *v*_*b*_.

## Conclusion

Our results show good agreement between the median values obtained by histology and the BPCA MRI methods and the 2CXM LMCA approach. However, the ETM yielded results that were completely different from the histological *v*_*b*_ values. Using BPCA methods for measuring blood volume has the crucial advantage of simple implementation, very short acquisition times, and a stable and accurate measurement. While LMCA models provide excellent curve fits and can in principle determine more physiological parameters than BPCA methods, they yield more inaccurate individual results.

## Supporting Information

S1 File Histological Data. (XLSX)

S2 File AIF and tissue data. The files contain the time points and the relaxation rate changes of the AIF and the five skeletal muscle regions of the pigs hind limb.
(ZIP)
